# Antitrypanosomal Activity of *Anthriscus* Nemorosa Essential Oils and Combinations of Their Main Constituents

**DOI:** 10.3390/antibiotics10111413

**Published:** 2021-11-18

**Authors:** Cecilia Baldassarri, Giulia Falappa, Eugenia Mazzara, Laura Acquaticci, Elena Ossoli, Diego Romano Perinelli, Giulia Bonacucina, Stefano Dall’Acqua, Loredana Cappellacci, Filippo Maggi, Farahnaz Ranjbarian, Anders Hofer, Riccardo Petrelli

**Affiliations:** 1 Chemistry Interdisciplinary Project (CHIP) via Madonna delle Carceri, School of Pharmacy, University of Camerino, 62032 Camerino, Italy; cecilia.baldassarri@unicam.it (C.B.); giulia.falappa@studenti.unicam.it (G.F.); eugenia.mazzara@unicam.it (E.M.); laura.acquaticci@unicam.it (L.A.); elena.ossoli@unicam.it (E.O.); diego.perinelli@unicam.it (D.R.P.); giulia.bonacucina@unicam.it (G.B.); loredana.cappellacci@unicam.it (L.C.); filippo.maggi@unicam.it (F.M.); 2 Natural Product Laboratory, Department of Pharmaceutical and Pharmacological Sciences, University of Padova, 35131 Padova, Italy; stefano.dallacqua@unipd.it; 3 Department of Medical Biochemistry and Biophysics, Umeå University, 90736 Umeå, Sweden; farahnaz.ranjbarian@umu.se

**Keywords:** *A. nemorosa*, *Trypanosoma brucei*, farnesene, nanoemulsions, artificial mixtures

## Abstract

This study aimed to investigate the susceptibility of *Trypanosoma brucei* to the *Anthriscus nemorosa* essential oils (EOs), isolated compounds from these oils, and artificial mixtures of the isolated compounds in their conventional and nanoencapsulated forms. The chemical composition of the essential oils from the aerial parts and roots of *Anthriscus nemorosa*, obtained from a wild population growing in central Italy, were analyzed by gas chromatography/mass spectrometry (GC/MS). In both cases, the predominant class of compounds was monoterpene hydrocarbons, which were more abundant in the EOs from the roots (81.5%) than the aerial parts (74.0%). The overall results of this work have shed light on the biological properties of *A. nemorosa* EO from aerial parts (EC_50_ = 1.17 μg/mL), farnesene (EC_50_ = 0.84 μg/mL), and artificial mixtures (Mix 3–5, EC_50_ in the range of 1.27 to 1.58 μg/mL) as relevant sources of antiprotozoal substances. Furthermore, the pool measurements of ADP (adenosine diphosphate) and NTPs (nucleoside triphosphates) in the cultivated bloodstream form of trypanosomes exposed to different concentrations of EOs showed a disturbed energy metabolism, as indicated by increased pools of ADP in comparison to ATP (adenosine triphosphate) and other NTPs. Ultimately, this study highlights the significant efficacy of *A. nemorosa* EO to develop long-lasting and effective antiprotozoal formulations, including nanoemulsions.

## 1. Introduction

African trypanosomiasis (HAT), also called sleeping sickness, is an endemic disease of rural sub-Saharan Africa, caused by two species of *Trypanosoma brucei*: *T. brucei gambiense* and *T. brucei rhodesiense* [1]. The *T. brucei gambiense* parasite causes more than 98% of human infections, and it is mainly present in Western and Central Africa. In contrast, the *T. brucei rhodesiense* parasite mostly affects animals and rarely humans, and is most common in eastern and southern Africa [2]. The tsetse fly mediates the transmission of the parasite causing the disease. *T. brucei gambiense* infections are chronic, and the slow progression of the disease allows the patients to survive for several years. In contrast, *T. brucei rhodesiense* infections are acute, and the faster progression of the disease leads to death within a few months if untreated [3,4]. Two stages characterize HAT: the first stage symptoms are fever, headache, joint pain, and itching due to the parasite’s passage in the blood and lymph system [3,4]. The second stage symptoms are poor coordination, dramatic mood swings, confusion, and convulsions due to the parasite’s passage in the central nervous system [3,4]. Gambiense HAT is treated with intramuscular pentamidine once a day for seven days in the early stage and with nifurtimox-eflornithine in the second stage with the hospitalization of the patients. Fexinidazole was introduced in 2018 as an oral treatment of gambiense HAT, and its efficacy was demonstrated in both stages of the disease. Therapy with fexinidazole is not conclusive for patients with severe central nervous system symptoms, and the bioavailability of the drug decreases dramatically if taken without a meal with subsequent failure of the therapy [5]. Nowadays, the development of new therapeutic strategies for HAT treatment represents an enormous challenge in terms of toxicity, costs, and administration, especially in underdeveloped countries. In this regard, natural products, with particular attention to essential oils (EOs), might represent useful tools in discovering new promising drug candidates. In particular, plants from the Apiaceae family have been investigated for their promising activity against *T. brucei* [6,7]. Within this family, *Anthriscus nemorosa* (M.Bieb.) Spreng. is an aromatic plant, which is also known with the names of ‘gimigimi’ or ‘peçek’ in Turkey [8]. The plant grows in Europe and Asia’s temperate areas, in countries like Italy and Japan [9]. The traditional medicine of several countries reserves ample space for *A. nemorosa* for the treatment of several diseases. For instance, its fruits are traditionally used as a remedy for gastrointestinal disorders [8], and for treating inflammation and rheumatism [10,11]. The plant is also used to prepare herby cheese, called ‘otlu peynir’, in different areas of Turkey [12]. Moreover, ethnobotanical studies revealed its usefulness to improve memory in Alzheimer’s disease. This plant also holds several pharmacological activities, as preventing anxiety and depression [12,13,14]. The root EO and the flower ethyl acetate extract showed a significant antioxidant activity [12]. *A. nemorosa* also exhibits an antifungal potential, and the hexane extract of its aerial parts was highly efficient against *Candida albicans* [15]. As far as the volatile fraction is concerned, only a few studies are available on the chemical composition. Because EOs of some Apiaceae plants represent an attractive alternative approach to fighting protozoan parasites, this study aimed to better define the chemical composition of *A. nemorosa* EOs and to evaluate their antitrypanosomal activity against *T. brucei*. Being naturally occurring compounds, EOs and their components are always believed to be environmentally safe, user-friendly, and generally not toxic to humans [12]. Unfortunately, EOs can hide unexpected drawbacks (e.g., low stability and water solubility, high volatility, and side-effects). To overcome these issues, we here used encapsulation in nanoemulsions to improve the chemical-physical profile of the EOs.

## 2. Results

### 2.1. EO Compositions

Chemical compositions of the EOs hydrodistilled from the *A. nemorosa* aerial parts (*A. nemorosa* EO-AP) and roots (*A. nemorosa* EO-R) are reported in Table 1. Thirty-four volatile components were identified in the two EO samples by GC-MS, accounting for 98.1 and 98.6% of the total compositions, respectively. In both cases, the predominant class of compounds was monoterpene hydrocarbons, which were more abundant in the EOs from the roots (81.5%) than the aerial parts (74.0%). Among the monoterpene hydrocarbons, myrcene dominated over the others, especially in the aerial parts, accounting for 40.1 and 19.9% of the chemical profile in the aerial parts and roots, respectively. In contrast, the β-pinene content in the *A. nemorosa* EO-AP was only 3.0%, but it represented 22.0% of the total composition in the *A. nemorosa* EO-R. γ—Terpinene was detected at 11.6 and 9.2% in EOs from aerial parts and roots, respectively, while (Z)-β-ocimene was more abundant in the EO from roots (8.9%) than the aerial parts (4.4%). Another group of compounds occurring in noteworthy levels in the two EOs was the sesquiterpene hydrocarbons. They accounted for only 1% of the whole composition in the case of the root EO, while they represented 18.8% of the chemical profile in the aerial part EO, with germacrene D (10.4%), (*E*,*E*)-α-farnesene (4.0%) and δ-cadinene (2.3%) as the main constituents. It is interesting to note that the class of oxygenated monoterpenes was totally absent in both EOs. In contrast, oxygenated sesquiterpenes were detected at 3.0 and 4.4% in the EOs from aerial parts and roots, respectively. Moreover, other compounds, such as alkanes, were found in the EOs from the aerial parts and roots (2.3 and 11.2%, respectively), with nonane being the most significant one (1.7 and 9.5% in the EOs from aerial parts and roots, respectively).

### 2.2. Antitrypanosomal Activity

**Figure 1:**
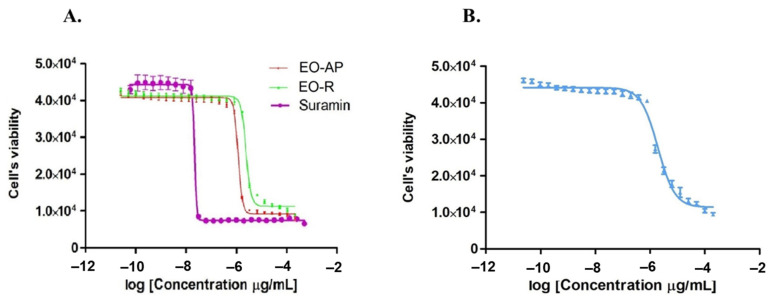
Growth inhibition of *T. brucei* s427 cells by *A. nemorosa* EO from aerial parts (EO-AP) and roots (EO-R) and by farnesene. (**A**). Growth inhibition of *T. brucei* treated with EO-AP (red curve) and EO-R (green curve) with Suramin as control (purple curve). (**B**). *T. brucei* growth inhibition with farnesene.

**Figure 2:**
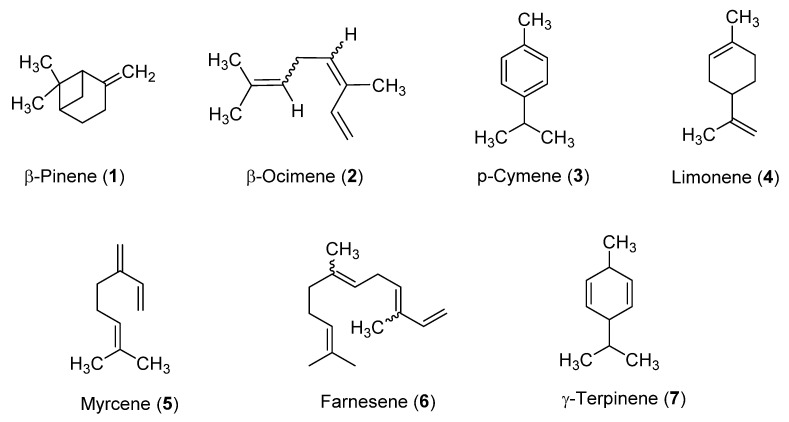
Chemical structures of (-)-β-pinene (**1**), β-ocimene (**2**), *p*-cymene (**3**), limonene (**4**), myrcene (**5**), farnesene (**6**), and γ-terpinene (**7**).

Testing the EOs hydrodistilled from *A. nemorosa* aerial parts and roots, we showed that both of them effectively inhibited *T. brucei* proliferation, especially the EO from aerial parts with an EC_50_ value of 1.17 μg/mL (Table 2 and Figure 1) and a selective index (SI) of 4.65. The root EO was two times less active (EC_50_ = 2.36 μg/mL) and selective (SI = 2.25). Since the aerial part EO was the most active of the two, it was chosen for further investigations. The major components found in the *A. nemorosa* EO (aerial parts) belong to two chemical groups, namely monoterpene hydrocarbons and sesquiterpene hydrocarbons. Among the constituents tested, farnesene (EC_50_ = 0.84 µg/mL) and β-ocimene (EC_50_ = 1.1 µg/mL) were the most potent and showed EC_50_ values slightly lower than that of the intact Isocratic HPLC analysis for the simultaneous determination of dNTPs, rNTPs, and ADP in biological samples oil. The β-ocimene tested was then a mixture of *Z* and *E* isomers. β-pinene, *p*-cymene, limonene, myrcene, and γ-terpinene were less active and had EC_50_ values higher than 4.5 μg/mL. The sesquiterpene hydrocarbon germacrene D was not tested because it is not commercially available. As reported in Table 2, the antitrypanosomal activity of the major compounds assayed was in the order: farnesene > β-ocimene > *p*-cymene > limonene > β-pinene > myrcene > γ-terpinene. The chemical structures of the bioactive compounds varied from linear (farnesene, β-ocimene, and myrcene) to monocyclic (limonene, *p*-cymene, and γ-terpinene) and bicyclic derivatives (β-pinene), and contain one to four unsaturations (Figure 2).

### 2.3. Contribution of Individual Compounds to the Overall Antitrypanosomal Activity and Synergistic Effects

**Figure 3:**
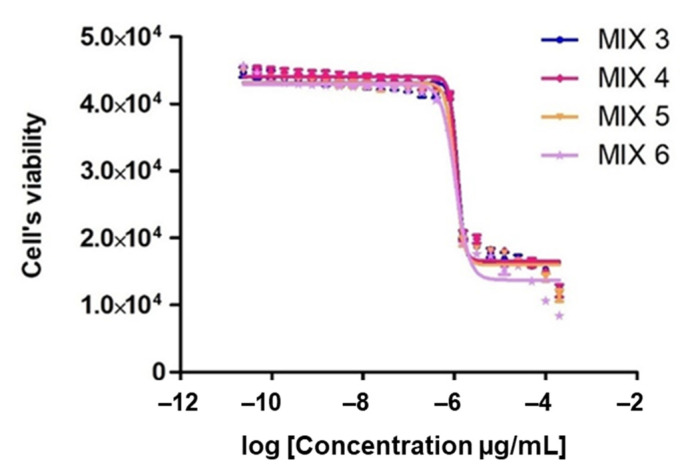
Tests of combinations of EO constituents against *T. brucei*. The different mixes are described in Table 3 and the curves are colored according to the following scheme: Mix 3 (blue curve), Mix 4 (red curve), Mix 5 (orange curve), and Mix 6 (purple curve). Each EC_50_ value represents the mean of four independent experiments with standard errors.

In order to better understand synergistic effects of the different components in the *A. nemorosa* EO-AP, we carried out a compound elimination assay where omitted compounds were replaced by DMSO (Table 3 and Figure 3). It then emerged that limonene, β-ocimene, *p*-cymene, and farnesene are the EO’s major antitrypanosomal constituents whereas myrcene seemed to have a protective effect (Table 3). The protective effect was evident from that the removal of myrcene led to a decrease in the EC_50_ value from 7.25 to 2.25 μg/mL, and the antitrypanosomal effect of limonene, β-ocimene, *p*-cymene, and farnesene was evident from that if three of these components were removed (Mix 8–11), it led to an increased EC_50_ value. The strongest effect was if limonene, β-ocimene, and farnesene were simultaneously removed from the mixture leading to an EC_50_ increase from 7.25 (Mix 1) to 49.2 μg/mL (Mix 9).

In Table 3, Wadley’s formula was used to analyze synergistic interactions among some of the constituents of the *A. nemorosa* EO-AP. The different constituents displayed additive, antagonistic, and synergistic relationships. Our results indicated several additive interactions between the six main components of *A. nemorosa* EO-AP (Mix 1, Mix 8–9, and Mix 11). The simultaneous combination of the six major constituents (Mix 1) produced an EC_50_ value (7.25 μg/mL), which is 1.7 fold higher than expected (4.09 μg/mL, R = 0.56, additive). On the other hand, limonene, β-ocimene, *p*-cymene, and farnesene all showed synergistic effects with each other (Mix 3–7), and this synergy was prevented if myrcene was present (Mix 1). In contrast, there was no negative effect of removing just one of these four active compounds (compare Mix 4–7 with Mix 3), indicating that the synergy is not dependent on that all four components are present.

### 2.4. Preparation and Characterization of A. nemorosa EO-Based Nanoemulsions

To investigate whether the antitrypanosomal activity and selective index could be improved, nanoemulsions of *A. nemorosa* EO-AP were formulated and tested against *T. brucei* and mammalian cells. The surfactant polysorbate 80 at a concentration of 2% (*w/w*) was used as the stabilizing agent in these nanoemulsions (Table 4). *A. nemorosa* EO-based nanoemulsions were formulated to evaluate the influence of the nanoencapsulation on the active ingredients’ biological activity. An initial screening to assess the *A. nemorosa* EO-AP total oily phase and the surfactant percentages was carried out to select the final quali-quantitative composition of nanoemulsions named NANO A and NANO B (Table 4).

**Figure 4:**
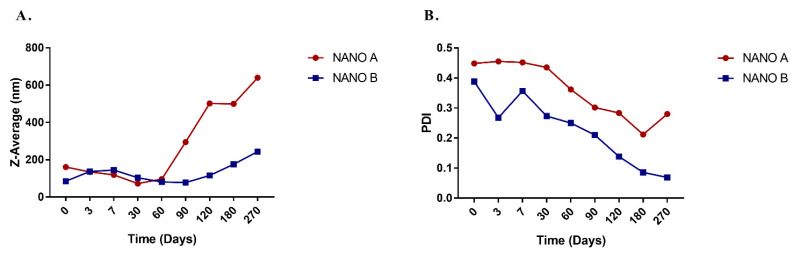
Mean droplet size (**A**) and polydispersity index (**B**) of *A. nemorosa* EO-based nanoemulsions at different time points. The mean droplet size is indicated as Z-Average with the diameter in nm.

The obtained nanoemulsified systems were assessed by the absence of visible oily droplets through optical microscopy. The droplet size distributions of nanoemulsions were centered between 140 and 170 nm (mean diameter, Z-average) up to two months from the preparation for NANO A and up to four months for NANO B. At a longer storage time, both nanoemulsions showed an increase in the mean droplet diameter, which was more pronounced for NANO A than NANO B. Indeed, the mean droplet diameter reached approximately 700 nm for NANO A (after 9 months from the preparation), while it was below 300 nm for NANO B (Figure 4A). Besides, the polydispersity Index (PDI) values were lower for NANO B than NANO A (Figure 4B). PDI is an indicator (ranging from 0 to 1) of the width of the distribution (a smaller index indicates a monodispersed system) [19]. Overall, these results highlight the role of ethyl oleate as co-solvent in the formulation, since it has improved the particle size and stability of nanoemulsions.

To determine their in vitro antitrypanosomal activity and selectivity, NANO A and NANO B were tested on *T. brucei* s427 and Balb/3T3 cells with the same procedure used for EOs, pure compounds, and artificial mixtures. The EC_50_ values of NANO A and NANO B were much higher than those obtained from testing the pure *A. nemorosa* EO-AP, mainly because the nanoemulsions only contain 6% *w/w* of EO and the major fraction of the weight is water. We have therefore also included EC_50_ values normalized by the percentage of EO in the nanoemulsions in brackets. These normalized values in Table 5 are directly comparable to the EO-AP EC_50_ value reported in Table 1. The normalized EC_50_ values are still higher than the EC_50_ obtained with the EO itself, but on the other hand the nanoemulsions is also less toxic to mammalian cells (Table 5). Thus, an interpretation of the results is that the nanoemulsions prevent the effect of the EO on both the trypanosomes and on the mammalian cells but that this ameliorating effect is stronger on the mammalian cells, especially with NANO A, resulting in a two times higher selectivity index compared to the EO itself.

### 2.5. Determination of NTP and ADP Pools in T. brucei

**Figure 5:**
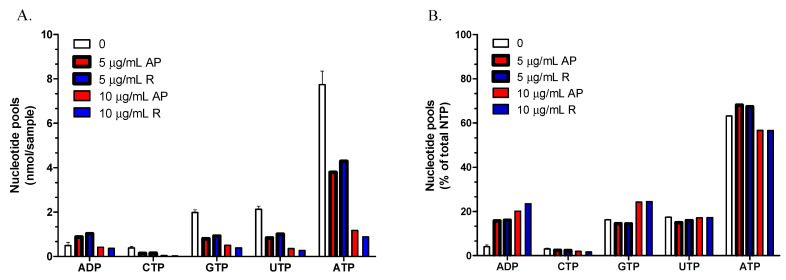
Effect of *A. nemorosa* EOs on *T. brucei* nucleotide pools. (**A**). Levels in nmol per sample of nucleoside triphosphates (NTPs) and ADP when *T. brucei* cells are treated with different concentrations of EOs from *A. nemorosa* aerial parts (AP) and roots (R); (**B**). Same data as in A, but the nucleotides are replotted as a percentage of the total NTP pool.

Finally, we analyzed the effect of *A. nemorosa* EOs on nucleotide pools in *T. brucei* grown in culture. Extracted nucleotides from the trypanosomes were separated by HPLC for quantification. The treatment of trypanosomes with different concentrations of *A. nemorosa* EOs (0–10 μg/mL) led to a concentration-dependent decrease of all NTP pools (Figure 5A). The four NTPs are equally affected at the lower EO concentration, and only some deviation is observed at the higher dose. This is most clearly seen in Figure 5B where relative nucleotide pools are plotted as a percentage of the total NTP pool. In contrast, the relative ADP pools are strongly affected by the treatment indicating a disturbed cellular energy charge (ATP: ADP ratio). The effects on the *T. brucei* nucleotide pools were similar with the EOs from aerial parts and roots.

## 3. Discussion

The EOs and their isolated components provide evolutionary validated core structures that can inspire the synthesis of new chemotherapeutic agents. Due to the singular chemical features of their main constituents (e.g., small lipophilic molecules), EOs can cross the cellular membrane and interact with intracellular targets, playing essential roles against pathogen survival, proliferation, and differentiation [20]. The chemical compositions of the EOs obtained from the aerial parts and roots of a central Italian population of *A. nemorosa* vary considerably from those previously reported for this species. Karakaya et al. found high levels of spathulenol (49.6%) and biclycogermacrene (8.9%) and almost a complete absence of myrcene in the aerial parts of a Turkish population [12]. Nickavar et al. studied an Iranian population and found (*E*)-nerolidol (41.7%), β-elemene (13%), and α-zingiberene (9.9%) as the major constituents of the aerial parts of *A. nemorosa* [21]. (E)-caryophyllene (23.6%), caryophyllene oxide (12.3%), δ-cadinene (12.1%), and *trans*-pinocarveol (9.8%) were identified as major constituents of aerial parts EO of *A. nemorosa* from another Turkish population [13]. Lastly, Kiliç et al. reported that (E)-caryophyllene (15.8%), caryophyllene oxide (14.5%), δ-cadinene (13.4%), germacrene D (8.9%), and *trans*-pinocarveol (6.2%) are the primal components of aerial parts EO of *A. nemorosa* from Turkey [22]. Considering our results, we believe that different factors such as geographic origin, genetics, period of harvesting and environmental conditions, type of processing, and drying may affect the observed variability.

The first important outcome that emerged from our study is linked to the relevant antitrypanosomal activity showed by the *A. nemorosa* EO with EC_50_ values of 1.17 μg/mL (SI = 4.65) and 2.36 μg/mL (SI = 2.25) for aerial parts and roots, respectively (Table 2). Plant EOs frequently exhibit stronger antitrypanosomal activity in comparison to each of their individual constituents. However, in this case, the major components of the EO had only a modest antitrypanosomal effect. Instead, the major activity came from two less abundant components, farnesene and β-ocimene, which when assessed separately showed equal or greater antitrypanosomal effects compared to the natural *A. nemorosa* EO-AP. Farnesene had a lower EC_50_ value (0.85 µg/mL) compared both to the intact oil (1.17 µg/mL) and the best artificial mixture (1.06 µg/mL for Mix 6, Table 2). The higher activity of farnesene is logical because it is the strongest antitrypanosomal agent in the mixture and is more concentrated in its pure form. On the contrary, it is remarkable that the EO gives almost as high effect (low EC_50_) as farnesene itself, despite that the two major antitrypanosomal agents, farnesene and β-ocimene, together make up only 11.9% of the total EO. This is a main reason why we investigated synergistic interactions in the EO.

Our results demonstrate additive, synergistic, and antagonistic interactions among constituents of *A. nemorosa* EO from aerial parts. Using Wadley’s model, we compared the calculated EC_50_ values of our artificial EOs with expected EC_50_ values. To determine the potential contribution of each component to the overall toxicity of the EO, we made a blend of all major constituents (Mix 1), mimicking the natural EO but excluding components with no antitrypanosomal activity. We also prepared different blends, each lacking one or several major constituents being replaced by DMSO (Mix 2–11), relying on the natural composition of aerial part EO as indicated by GC-MS. When we mixed the six constituents (Mix 1), we found that their observed antitrypanosomal activity was not as high as expected (EC_50_ = 7.25 µg/mL of Mix 1 vs. EC_50_ = 1.17 µg/mL of the corresponding EO), and the relationship between the constituents of the mixture was additive (R = 0.56). Presumably, the inactive constituents removed from Mix 1 have some additional synergistic effects on the six components. Although not active individually, their presence is necessary to achieve the observed antitrypanosomal activity. As shown in Table 3, the activity of *A. nemorosa* EO from aerial parts might be attributed to the presence of its bioactive constituents, limonene, β-ocimene, *p*-cymene, and farnesene (5.7%, 7.2%, 2.2%, and 4.7% in the natural EO and 9.1%, 11.4%, 3.7% and 7.4% in the artificial EO, respectively). Different blends of these components (Mix 3–7 in Table 3) showed strong synergy with R values between 3–6. Interestingly, this synergy was weakened in the presence of β-pinene and disappeared completely in the presence of myrcene. Considering the concentrations of those three components in the essential oil of aerial parts (19.8% combined) and their enhanced effect when combined, the antitrypanosomal activity of *A. nemorosa* EO-AP might mainly be a consequence of their synergistic interaction. In the EO itself, the antagonistic effects from myrcene and β-pinene seems not to be present and the EC_50_ value is comparable to Mix 3 where only the synergistic components are present (EC_50_ values: 1.17 µg/mL for the EO vs. 1.43 µg/mL for Mix 3).

The synergistic interactions of EO components might be useful in two different scenarios. First, using artificial mixtures based on EO composition rather than single isolated components might be a more powerful multitarget strategy for tackling trypanosomiasis. Second, understanding the mechanism behind the synergistic interactions between EO components could further help to develop more efficacious antitrypanosomal drugs based on custom blends of monoterpenoids/sesquiterpenoids that do not occur in nature. Various mechanisms are triggering synergistic interactions [23]. The potential synergistic interaction detected in our study may be caused by one or more of these proposed mechanisms because the *A. nemorosa* EO-AP is a complex mixture of components. For instance, monoterpenes like limonene, characterized by a cycle bearing an exocyclic methylene group, can react with functional groups of proteins, such as trypanothione synthase, producing cell oxidative damage [24]. The aromatic monoterpene *p*-cymene, being highly hydrophobic, is easily incorporated into the lipid bilayer, facilitating other bioactive components’ penetration. A previous study conducted by Monzote et al. has highlighted the antiprotozoal properties of *p*-cymene [25]. The acyclic monoterpene hydrocarbon β-ocimene, is instead involved in the plant defense against predators and is responsible for evoking specific responses [26]. The first evidence of its effect against *T. brucei* was reported in 2018 (EC_50_ of 1.1 μg/mL and SI > 91) [6]. Lastly, the acyclic olefin (E)-β-farnesene is an efficient and robust alarm pheromone in most aphid species. Various plants also produce (E)-β-Farnesene as aphid repellant since aphids exposed to this chemical become agitated and disperse from the host. Although (E)-β-farnesene is widespread in plants, this is the first report about its effects against *T. brucei* (EC_50_ = 0.84 µg/mL).

Generally, EOs show many biological activities, although their use is often limited by several drawbacks (i.e., polymerization, isomerization, oxidation, and rearrangement), which depend on several environmental parameters. These instability issues may end up in a reduction or loss of activity. Moreover, EOs have poor physicochemical properties (e.g., water insolubility, high volatility, and a short half-life) that make them difficult to handle. In this regard, nanoemulsions can help to overcome the drawbacks of EOs by improving their physicochemical properties and stability. Moreover, due to their subcellular size, nanoemulsions could enhance the bioavailability and active compounds effectiveness through their solubilization into tiny oily droplets, thereby allowing a deeper tissue penetration and easier cellular uptake. Previous studies highlight the importance of vehiculation of EOs against parasites of medical importance, including *Plasmodium*, *Leishmania*, and *Trypanosoma*, as well as against vectors of human diseases. For instance, in a study carried out by Bouyahya et al. in 2017, the nanoemulsions of *Lavandula angustifolia* L. and *Rosmarinus officinalis* L. EOs showed antiparasitic effects greater than that obtained with the non-emulsified EOs [27]. As can been seen in Table 5, the EC_50_ values obtained with the nanoemulsions are higher than with the *A. nemorosa* EO aerial parts. On the other hand, the mammalian control cell lines were also less sensitive giving a slightly better selective index. In fact, the selectivity index against the trypanosomes in comparison to the mammalian cells was better with NANO A than with the *A. nemorosa* EO-AP (SI = 8.42 for NANO A and 4.65 for the *A. nemorosa* EO-AP). The surfactant itself that was used to make the nanoemulsions (polysorbate 80) was inactive in the concentration range tested.

To hypothesize a potential mechanism of action of *A. nemorosa* EO-AP and *A. nemorosa* EO-R, NTP and ADP pools were measured by HPLC. As shown in Figure 5, the treatment of the parasites led to a general decrease in the NTP pools and increased relative ADP levels. The increased ADP levels is a sign of disturbed energy metabolism, indicating that the cell is not able to phosphorylate ADP to ATP efficiently. A consequence of the decreased ATP pools is that other NTP pools also decrease because they are metabolically linked to each other via NDP kinase and other enzymes. NDP kinase uses NTPs to phosphorylate NDPs. In a normal situation, it will most often use ATP as a phosphate donor because it has the highest concentration in the cell, but it can also use other NTPs depending on their abundance. The decreased pools of other NTP pools is therefore also likely to be a consequence of the disturbed energy metabolism. Another factor that can affect the measured nucleotide levels is that the cells are dying and that the number of living cells is, therefore, less in the end of the incubation period as compared to the untreated cells. We have tried to minimize this factor by keeping the incubation period short. However, with the higher dose of EO, the NTP pools were decreased even more, but with no further increase in the ADP pools indicating that cell death can be a contributing factor also in this short time frame if the EO concentration is high enough. A disturbed energy metabolism is very harmful to the cell, which needs ATP to keep intracellular ion concentrations intact.

## 4. Materials and Methods

### 4.1. Plant Material

Aerial parts and roots of a wild population of *A. nemorosa* growing in Camerino, central Italy (620 m a.s.l., N 43°1′3.78″; E 13°06′9.68″) were collected in May 2019. A voucher specimen was authenticated by one of the authors (F.M.) and deposited in the *Herbarium Universitatis Camerinensis* (CAME, included in the online edition of Index Herbariorum c/o School of Biosciences and Veterinary Medicine, University of Camerino, Italy) under the code CAME#29276; it was also archived in the anArchive system for Botanical Data (http://www.anarchive.it) (accessed on 27 October 2021).

### 4.2. Chemicals

Pure constituents of the EOs were purchased from Sigma-Aldrich [β-pinene (99%), farnesene (a mixture of isomers), limonene (97%), ocimene (a mixture of α and β isomers), *p*-cymene (99%), γ-terpinene (97%), terpinolene (85%), St Louis, MO, USA], and Merck (myrcene; Darmstadt, Germany).

### 4.3. Hydrodistillation

Hydrodistillation (HD) was performed on dried samples of aerial parts and roots of *A. nemorosa*. In both cases, we used 2.5 kg of plant material soaked in a 20 L glass flask filled with 12 L of water, heated by a mantle system Falc MA (Falc Instruments, Treviglio, Italy) for 5 h. The EOs were recovered by a glass Clevenger-type apparatus and stored on the fridge before chemical characterization and biological evaluation. The EO yield was calculated on a dry-weight basis. 

### 4.4. GC-MS Analysis

The chemical composition of *A. nemorosa* EOs was analyzed through an Agilent 6890N GC-MS system coupled to a 5973N single quadrupole detector mass spectrometer. The separation was provided by an HP-5MS capillary column (5% phenylmethylpolysiloxane, 30 m × 0.25 mm i.d., 0.1 μm f.t., Agilent, Santa Clara, CA, USA). The temperature program was as follows: 60 °C for 5 min, then 4 °C min^−^^1^ up to 220 °C, finally 11 °C min^−^^1^ to 280 °C, maintained for 15 min, for a total run time of 65 min. The temperature of the injector and detector was 280 °C. Helium (He) was the carrier gas, with a flow rate of 1 mL min^−^^1^ and a 1:50 split ratio. The chromatograms were acquired in full scan in the range 29.0–400.0 uma, using electron-impact (EI, 70 eV) mode. Dilution 1:100 of essential oils in n-hexane was injected (2 μL) into the GC-MS system. The MSD ChemStation (Agilent, Santa Clara, CA, USA, Version G1701DA D.01.00) and the NIST Mass Spectral Search Program were employed for the data analysis. Identifying the principal compounds was achieved by the correspondence of retention indices and mass spectra to those of ADAMS, NIST 17, FFNSC2, and WILEY 275 libraries. Furthermore, the analytical standards available in the laboratory (Sigma-Aldrich, Milan, Italy) were used for further confirmation. The relative peak area percentages were obtained by area normalization without using correction factors [28].

### 4.5. T. brucei and Mammalian Cell Culture

*T. brucei* s427 bloodstream forms (subspecies *T. b. brucei*) and mammalian Balb/3T3 fibroblasts (ATCC no CCL-163) were harvested as reported in Ngahang Kamte et al. [17]. *T. brucei* cells were cultured in HMI-9 medium supplemented with 5% (*v/v*) heat inactivated fetal bovine serum (FBS) and 10 mL/L of 100× penicillin-streptomycin (Gibco, Billings, MT, USA) at a temperature of 37 °C with 5% CO_2_.

Mammalian fibroblasts were cultured in Dulbecco’s modified Eagle’s medium (Sigma-Aldrich, St Louis, MO, USA) supplemented with 10% (*v/v*) heat-inactivated fetal bovine serum, L-glutamine (0.584 g/L) and 10 mL/L of 100× penicillin-streptomycin (Gibco, Billings, MT, USA) with the same temperature and CO_2_ conditions as the parasites.

### 4.6. Growth Inhibition Assay on T. brucei and Balb/3T3 Cells

All the compounds that were tested were dissolved in dimethyl sulfoxide (DMSO). The final DMSO concentration in each well was less than 1% in all experiments and has no effect on the trypanosomes in this concentration range. They were serially diluted in 96-wells microtiter plates with growth medium to obtain a range of concentrations from 2 × 10^−5^ µg/mL to 200 µg/mL (100 µL/well). In each well 100 µL of *T. brucei* or mammalian fibroblasts cell culture were added to have 20,000 and 2000 cells per well, respectively. The plates were incubated for 48 h at 37 °C with 5% CO_2_, treated with 20 µL of 0.5 mM resazurine (Sigma-Aldrich, St Louis, MO, USA) and treated for 24 h at the incubation conditions. Subsequently, the surviving cells were quantified by fluorescence (540 nm excitation and 590 nm emission) with an Infinite M200 microplate reader (Tecan Group, Ltd. Männedorf, Switzerland). Data were analyzed using the GraphPad Prism 5.04 software to obtain EC_50_ values by fitting the data to a log inhibitor vs. response curve (variable slope, four parameters) and calculating the selectivity index (SI) from the comparison of *T. brucei* and Balb/3T3 EC_50_ values.

### 4.7. Comparative Activities

To evaluate individual constituents’ contribution to overall activity, a compound-elimination assay was conducted as previously described by Miresmailli [29]. A series of artificial EOs were prepared using the constituents in their natural proportions, either as the full mixture or with one, two, or three constituents omitted. Each artificial EO had the same amounts in weight of individual compounds as in the complete EO, with the amount of missing compounds replaced by DMSO.

### 4.8. Synergistic Interactions among Four Major Constituents

To evaluate potential synergies between the six major constituents (farnesene, β-ocimene, *p*-cymene, limonene, β-pinene, myrcene), mixtures were prepared following the actual constituent ratio based on chemical analysis of the EO. These artificial oils were applied to *T. brucei* or mammalian cell culture, and their EC_50_ values were calculated after 48 h. To determine the mixtures’ relationships, we used a statistical model, named Wadley’s model [30], to compare expected and observed EC_50_ values. Based on Wadley’s calculation, the expected EC_50_ values (assuming additive interaction) were determined from the equation:

$$E=\frac{a+b+c\ldots +n}{\frac{a}{{\mathrm{EC}}_{50\left(a\right)}}+\frac{b}{{\mathrm{EC}}_{50\left(b\right)}}+\frac{c}{{\mathrm{EC}}_{50\left(c\right)}}\ldots +\frac{n}{{\mathrm{EC}}_{50\left(n\right)}}}$$

where a  is the proportion of compound A in the mixture, and ${\mathrm{EC}}_{50\left(a\right)}$ is the EC_50_ of compound A. The interaction between the observed and theoretical EC_50_ values was compared as:

$$R=\frac{expected\;{\mathrm{EC}}_{50}}{observed\;{\mathrm{EC}}_{50}}$$

The relationship between the constituents of the mixture was defined as either synergistic (when R ˃ 1.5), additive (1.5 ≥R ˃ 0.5), or antagonistic (R ≤ 0.5) based on this model.

### 4.9. Preparation of Anthriscus nemorosa EO-Based Nanoemulsions

For the preparation of NANO A, *A. nemorosa* EO (from aerial parts) was added dropwise to a Polysorbate 80 aqueous solution under high-speed stirring (Ultraturrax T25 basic, IKA^®^ Werke GmbH & Co.KG, Staufen, Germany) for 5 min at 9500 rpm to form an emulsion with final concentrations of 6% (*w/w*) EO and 2% (*w/w*) polysorbate. The obtained emulsion was then homogenised by using a French Pressure Cell Press (American Instrument Company, Aminco, MY, USA) for four cycles at the pressure of 130 MPa. NANO B was formulated in a similar way but a 1:2 (*w/w*) mixture of ethyl oleate and EO was used in the first step instead of the pure EO.

### 4.10. Nanoemulsion Characterization

The formation of nanoemulsions was assessed by polarizing optical microscope (MT9000, Meiji Techno Co Ltd., Chikumazawa, Miyoshi machi, Iruma-gun, Saitama 354-0043, Japan) equipped with a 3-megapixel CMOS camera (Invenio 3S, DeltaPix, Denmark). Particle size measurements were performed through the dynamic light scattering (DLS) technique using a Zetasizer nanoS (Malvern Instruments, Worcestershire, UK) equipped with a backscattered light detector working at 173°. The analysis was performed at 25 °C at different time points: 0 day, 3 days, 7 days, 1 month, 2 months, 3 months, 4 months, 6 months, and 9 months [31].

### 4.11. Determination of NTP and ADP Pools in T. brucei

Blood-stream forms of *T. b. brucei* s427 were maintained at 37 °C and 5% CO_2_ in Hirumi’s modified Iscove’s Medium (HMI)-9 medium lacking thymidine and Serum Plus but containing 10% fetal bovine serum. The omission of thymidine does not affect the growth of the parasites [32]. Trypanosomes (50 mL, 10^6^ cells/mL), harvested in late logarithmic phase, were chilled on ice for 5 min before being collected and centrifuged at 3000× *g* for 5 min at 4 °C. Subsequently, the liquid was decanted and the pellet resuspended in the remaining liquid in the tube by tapping the bottom of the tube. The resuspended slurry was transferred to an Eppendorf tube, and centrifuged at 16,000× *g* for 1 min at 4 °C. The supernatant was discarded and the collected trypanosomes were disintegrated by pipetting them up and down in 500 µL of ice-cold 0.6 M trichloroacetic acid containing 15 mM MgCl_2_. The resultant solution was centrifuged at 16,000× *g* for 1 min at 4 °C, and the supernatant was extracted with 720 µL chloroform (78% *v/v*)-trioctylamine (22% *v/v*). Each sample was purified by an OASIS WAX cartridge as described previously [33] and the collected solution was evaporated to dryness in a Speedvac (Thermo Fisher Scientific Savant, Waltham, MA, USA) and dissolved in 200 µL of water. This fraction was analyzed by HPLC using the Fast Protocol [33] to quantify ADP and NTPs with a 150 × 3 mm C18-WP HPLC column (ChromaNik Technologies Inc., Osaka, Japan). The mobile phase was a ternary mixture of 43% solution A (23 g/L KH_2_PO_4_ pH 5.6 in 5.8% acetonitrile); 37% solution B (5.8% acetonitrile) and 20% solution C (3.52 g/L tetrabutylammonium bromide in 5.8% acetonitrile) and the column temperature was 30 °C. The nucleotides were quantified by measuring areas and comparing them to the areas of a 1 µM standard nucleotide mixture.

## 5. Conclusions

In conclusion, the overall results of our work have shed light on the biological properties of *A. nemorosa* EOs, farnesene and artificial mixtures (Mix 3–5 in Table 3) as relevant sources of bioactive substances and highlighted the opportunity to synthesize other farnesene-based derivatives as prototypes for future antiprotozoal drug development.

To our knowledge, this is the first report on the antiprotozoal activity of *A. nemorosa* EO and the sesquiterpene hydrocarbon farnesene. The EO had a strong effect on the *T. brucei* energy metabolism and caused a major decrease in NTP pools and a concomitant increase in ADP. However, further studies are needed to understand the more exact mode of action and the possible molecular targets related to the antitrypanosomal activity of *A. nemorosa* EOs, the artificial mixtures of EO components, and farnesene.

This study also highlights the significant efficacy of *A. nemorosa* EO nanoemulsions to develop long-lasting and effective prototype formulations active against *T. brucei*. The poor hygiene conditions of the affected geographic areas and the lack of proper facilities require straightforward and safe administration methods, and nanoemulsions represent one of these.

## Figures and Tables

**Table 1:** Chemical composition of the EOs obtained from *A. nemorosa* aerial parts and roots.

N°	Component ^a^	RI ^b^	RI Lit. ^c^	% *A. nemorosa* EO-AP	% *A. nemorosa* EO-R	ID ^d^
1	*n*-nonane	901	900	1.7	9.5	RI, MS
2	α-thujene	921	924	Tr ^e^	tr	RI, MS
3	α-pinene	927	932	0.4	1.7	Std
4	sabinene	964	969	0.4	0.4	Std
5	β-pinene	969	974	3.0	22.0	Std
6	myrcene	990	988	40.1	19.9	Std
7	*n*-decane	1001	1000	0.1	0.8	RI, MS
8	α-phellandrene	1004	1002		0.2	Std
9	α-terpinene	1015	1014	0.1	tr	Std
10	*p*-cymene	1022	1020	2.2	2.8	Std
11	limonene	1024	1024	5.7	11.5	Std
12	β-phellandrene	1026	1025	3.0	2.7	Std
13	(*Z*)-β-ocimene	1038	1032	4.4	8.9	Std
14	(*E*)-β-ocimene	1048	1044	2.8	1.8	Std
15	γ-terpinene	1056	1055	11.6	9.2	Std
16	*n*-undecane	1101	1100	0.4	0.9	RI, MS
17	*allo*-ocimene	1129	1128	0.2	0.5	RI, MS
18	β-elemene	1384	1389	0.4	tr	RI, MS
19	(*E*)-caryophyllene	1387	1417	0.6	0.1	Std
20	β-copaene	1420	1430	0.5		RI, MS
21	(*E*)-β-farnesene	1457	1440	0.7		Std
22	germacrene D	1472	1484	10.4		RI, MS
23	1-dodecanol	1476	1469		0.5	RI, MS
24	*epi*-cubebol	1487	1493		0.3	RI, MS
25	(*E*,*E*)-α-farnesene	1508	1505	4.0		RI, MS
26	δ-cadinene	1517	1522	2.3	0.9	RI, MS
27	elemol	1543	1534		0.5	RI, MS
28	guaiol	1591	1600		0.8	RI, MS
29	10-*epi*-γ-eudesmol	1608	1622		0.5	RI, MS
30	*epi*-α-muurolol + *epi*-α-cadinol	1633	1640/1638	1.0	0.5	RI, MS
31	β-eudesmol	1639	1649	0.2	0.4	RI, MS
32	valerianol	1643	1656	0.1	0.4	RI, MS
33	α-cadinol	1646	1652	1.7	0.4	RI, MS
34	bulnesol	1659	1670		0.7	RI, MS
	Oil yield (%, *w/w*)			0.06	0.08	
	Total identified (%)			98.1	98.6	
	Grouped compunds (%)					
	Monoterpene hydrocarbons			74.0	81.5	
	Oxygenated monoterpenes					
	Sesquiterpenes hydrocarbons			18.8	1.0	
	Oxygenated sesquiterpenes			3.0	4.4	
	Alkanes			2.3	11.2	
	Others				0.5	

^a^ Order of elution is from an HP-5MS column (30 m × 0.25 mm, 0.1 µm). ^b^ Linear retention index according to Van den Dool and Kratz (1963) [16]. ^c^ RI taken from ADAMS and/or NIST 17 and FFNSC3 libraries. ^d^ Identification method: Std, comparison with the analytical standard; RI, coherence of the calculated RI with those stored in ADAMS, NIST 17, and FFNSC3 libraries. MS, mass spectrum overlapping with those recorded in ADAMS, NIST 17, WILEY 275, and FFNSC3 libraries. ^e^ Traces relative % < 0.1.

**Table 2:** Antitrypanosomal activity of *A. nemorosa* essential oils and pure compounds. For the pure compounds, their percentages in EOs from *A. nemorosa* aerial parts (AP) and roots (R) are also listed just after the names of the compounds.

Samples	EC_50_		Selectivity Index (SI)
	*T. brucei* (s427)	Balb3T3	
**Essential oils**	**μg/mL**	**μg/mL**	
A. nemorosa aerial parts	1.17 ± 0.09	5.48 ± 0.61	4.65
A. nemorosa roots	2.36 ± 0.09	5.33 ± 0.49	2.25
**Pure compounds**	**μg/mL** **(** **μM)**	**μg/mL** **(** **μM)**	
β-Pinene (**1**): 3% AP, 22% R	11.4 ± 2.6 (83.7) ^c^	>100	>8.77
β-Ocimene (**2**): 7,2% AP, 10.7% R	1.1 ± 0.5 (8) ^b^	>100	>91
*p*-Cymene (**3**): 2.2% AP, 2.8% R	4.5 ± 1.0 (33) ^b^	28 ± 7	6.2
Limonene (**4**): 5.7% AP, 11.5% R	5.6 ± 1.6 (41) ^a^	>100	>18
Myrcene (**5**): 40.1% AP, 19.9% R	26.2 ± 0.44 (193)	49.6 ± 0.64	1.9
Farnesene (**6**): 4.7% AP	0.84 ± 0.04 (4.11)	4.09 ± 0.39	4.85
γ-Terpinene (**7**): 11.6% AP, 9.2% R	>100	>100	-
**Reference drug**	**μg/mL** **(** **μM)**	**μg/mL** **(** **μM)**	
Suramin	0.025 ± 0.001 (0.0147)	>5	>262

^a^ Data from [17]. ^b^ Data from [6]. ^c^ Data from [18].

**Table 3:** Comparative antitrypanosomal activity of six major components of the EO from *A. nemorosa* aerial parts. Stock solutions were prepared of the complete mixture and different alternative mixtures where one or several components were replaced by DMSO.

	Ratio% *w/w*							Observed *T.b. brucei* EC_50_ (μg/mL)	Observed Balb3T3 EC_50_ (μg/mL)	Selectivity Index (SI)	Expected EC_50_ ^a^ (μg/mL)		
	Myrcene	β-Pinene	Limonene	β-Ocimene	*p*-Cymene	Farnesene	Carrier (Acetone)				Wadley ^b^	*R* ^c^	S ^d^
Mix 1	63.7	4.7	9.1	11.4	3.7	7.4	x	7.25 ± 0.26	28.3 ± 1.06	3.91	4.09	0.56	Add
Mix 2	x	4.7	9.1	11.4	3.7	7.4	63.7	2.25 ± 0.18	9.22 ± 0.67	4.11	4.54	2.01	Syn
Mix 3	x	x	9.1	11.4	3.7	7.4	68.4	1.43 ± 0.16	5.14 ± 0.36	3.63	4.63	3.23	Syn
Mix 4	x	x	x	11.4	3.7	7.4	77.5	1.58 ± 0.14	5.48 ± 0.31	3.47	5.00	3.17	Syn
Mix 5	x	x	9.1	11.4	3.7	x	75.8	1.27 ± 0.02	6.98 ± 0.85	5.46	7.80	6.14	Syn
Mix 6	x	x	9.1	11.4	x	7.4	72.1	1.06 ± 0.04	3.83 ± 0.19	3.62	4.81	4.53	Syn
Mix 7	x	x	9.1	x	3.7	7.4	79.8	1.40 ± 0.11	5.77 ± 0.15	4.11	8.88	6.34	Syn
Mix 8	63.7	4.7	x	x	x	7.4	24.2	15.3 ± 0.33	29.3 ± 3.42	1.91	8.58	0.56	Add
Mix 9	63.7	4.7	x	x	3.7	x	27.9	49.2 ± 3.71	66.8 ± 3.19	1.36	27.28	0.55	Add
Mix 10	63.7	4.7	x	11.4	x	x	20.2	27.1 ± 0.51	25.6 ± 1.21	0.94	7.57	0.28	Ant
Mix 11	63.7	4.7	9.1	x	x	x	22.5	24.3 ± 0.45	60.9 ± 2.87	2.51	22.4	0.92	Add

^a^ Expected EC_50_ based on Wadley’s calculation model. ^b^ Wadley’s calculation of expected EC50. ^c^ Synergy ratio from Wadley’s calculation. ^d^ Determination of the interaction of the mixture based on Wadley’s determination method: *R* ˃ 1.5, synergistic (Syn) interaction; 1.5 ≥ *R* ˃ 0.5, additive (Add) interaction; when *R* ≤ 0.5, antagonistic interaction.

**Table 4:** Composition of the formulated *A. Nemorosa* EO-based nanoemulsions (NANO A and NANO B) and control.

Samples	Composition
Control	Polysorbate 80 at 2% (*w/w*) in water
NANO A	2% (*w/w*) polysorbate 80 + 6% (*w/w*) *A. nemorosa* EO from aerial parts
NANO B	2% (*w/w*) polysorbate 80 + 4% (*w/w*) *A. nemorosa* EO from aerial parts + 2% (*w/w*) ethyl oleate

**Table 5:** Antitrypanosomal activity of *A.nemorosa* EO from aerial parts (EO-AP) and nanoemulsions using NANO A and NANO B.

	EC_50_		Selectivity Index (SI)
	*T. brucei* (s427)	Balb3T3	
**Nanoemulsions**	**μg/mL**	**μg/mL**	
Control	>40,000	>40,000	
NANO A	167.4 ± 12.8 (normalized 9.89 ± 0.797)	1399 ± 464	8.35
NANO B	322.3 ± 43.5 (normalized 12.7 ± 1.72)	1745 ± 347	5.41
